# Gastroprotective effect of Arabincoside B isolated from *Caralluma arabica* against ethanol-induced gastric injury via modulating oxidative stress/SP/NK-1R/NF-κB loop

**DOI:** 10.1007/s10787-025-01885-w

**Published:** 2025-08-07

**Authors:** Dalia E. Ali, Othman S. S. Al-Hawshabi, Sarah A. Abd El-Aal, Eman Sheta, Sherihan Salaheldin Abdelhamid Ibrahim, Amira A. El-Gazar, Essam Abdel-Sattar, Ghada M. Ragab

**Affiliations:** 1 Department of Pharmacognosy and Natural Products, Faculty of Pharmacy, Pharos University in Alexandria, Alexandria, Egypt; 2 Department of Biology, Faculty of Science, University of Aden, Aden, Yemen; 3 Department of Pharmacology and Toxicology, College of pharmacy, University of Kut, Wasit, 52001 Iraq; 4 Department of Pathology, Faculty of Medicine, Alexandria University, Alexandria, Egypt; 5 Department of Pharmacology and Therapeutics, Faculty of Pharmacy, Pharos University in Alexandria, Canal El- Mahmoudia Street, Smouha, Alexandria, Egypt; 6 Department of Pharmacology and Toxicology, Faculty of Pharmacy, October 6 University, Giza, 12585 Egypt; 7 Department of Pharmacognosy, Faculty of Pharmacy, Cairo University, El-Kasr El-Aini St, Cairo, 11562 Egypt; 8 Department of Pharmacology and Toxicology, Faculty of Pharmacy, Misr University for Science and Technology, Giza, 12585 Egypt

**Keywords:** *Caralluma arabica*, Arabincoside B, Gastric ulcer, MUC-6, SP/NK-1R, Anti-inflammatory

## Abstract

Gastric ulcer is a common gastrointestinal condition. Arabincoside B (AR-B), a pregnane glycoside isolated from the aerial parts of *Caralluma arabica*, shows multiple pharmacological effects. This study aimed to investigate the gastroprotective therapeutic effects of AR-B in ethanol-induced gastric injury in rats. Rats were divided as follows: Group I (NC): received 1 mL/day of normal saline; Group II (PC): received distilled water then 95% ethanol (1 mL per rat) after 1 h; Group III (FAM): received oral famotidine (20 mg/kg); Groups IV and V (AR-B groups): received 25 and 50 mg/kg of AR-B, respectively. Treatments (FAM or AR-B) were administered 1 h prior to ethanol. The rats were killed after 1 h after ethanol administration. Gross inspections as well as histopathological assessment of stomach tissues of untreated ethanol-only treated rats revealed major alterations as compared to those of normal rats. Pretreatment with AR-B showed enhancement in the gross and histological alterations. Moreover, AR-B at the dose of (50 mg/kg) possessed superior anti-inflammatory and antioxidant effects. This was confirmed by a significant lowering of the serum IL-6 and TNF-α levels, decreasing p-NF-κB gastric expression, decreasing MDA, and increasing GSH gastric levels. Furthermore, AR-B caused a significant increase in TFF-2 and MUC-6 stomach tissue expression, preserving the gastric mucosa. In addition, gastric expression of substance P and NK-1R was decreased, which participated in the reduction of inflammation. The current research highlights the gastroprotective impact of AR-B especially at a dose of (50 mg/kg), suggesting its future role in preventing recurrence in peptic ulcer patients.

**Supplementary Information:**

The online version contains supplementary material available at 10.1007/s10787-025-01885-w.

## Introduction

Gastric ulcers (GUs), also known as peptic ulcers, are one of the most serious gastrointestinal disorders and are becoming more common worldwide. The estimated prevalence of stomach ulcers in Western countries is 0.2–0.5%, but in Asian countries, it ranges from 2 to 3%. The recurrence rates might reach 60% (Wang et al. 2020). The imbalance between the stomach’s constructive (Mucin, peptides, prostaglandins, and blood flow) and destructive systems (stomach acid, pepsin, and *Helicobacter pylori*) causes GUs (Wasman et al. 2010; Al Batran et al. 2013). Moreover, excessive intake of nonsteroidal anti-inflammatory drugs (NSAIDs), alcohol consumption, bacterial infections, stress, and refluxed bile salts can lead to the development of GUs (Fornai et al. 2011).

The stomach mucosal barrier is mostly composed of gel-forming mucins (MUC5AC and MUC-6), which increase fluid viscosity (Hoffmann 2015). The trefoil factor (TFF) family includes three peptides (TFF1, 2, 3) (Clyne et al. 2004). Particularly, TFF-2 is found in the stomach, for protection against gastrointestinal injury. It has been shown that TFF-2 specifically partners with MUC-6 to strengthen the gastric mucosal barrier. Furthermore, TFF-2 could enhance mucosal repair by stimulating proliferation and reducing gastric acid production from parietal cells (Aziz et al. 2019).

Anti-ulcer conventional medications are effective but have multiple drawbacks, relapse after discontinuation, and tolerance development. Due to these limitations, there is a need for investigating new natural alternatives (Cicala et al. 2013; Kinoshita et al. 2018; Ali et al. 2024a, b).

The *Caralluma* genus is part of the Asclepiadoideae subfamily of the Apocynaceae family and is typically located in arid areas of Asia, Africa, and Europe (Malladi et al. 2018). Historically, several *Caralluma* species have been utilized to address ailments including cancer, diabetes, gastrointestinal diseases, rheumatism, and infections caused by malaria and trypanosomiasis (Dutt et al. 2012; Qayyum et al. 2018). Arabincoside B (AR-B), a pregnane glycoside, was identified in 2022 from the aerial portions of *C. arabica* by Abdel-Sattar et al. El-Shiekh et al. (2023) investigated the capacity of (AR-B) to withstand oxidative stress, inhibit inflammatory mediators, augment inflammatory inhibitors, and delay apoptosis in serum and lung tissues (Abdel-Sattar et al. 2022; El-Shiekh et al. 2023). The AR-B had also been demonstrated to promote wound healing, making it a potential veterinary wound dressing (Ali et al. 2024a, b).

Three species of genus *Caralluma* including *C. edulis*, *C. tuberculata*, and *C. umbellata* were traditionally used for the treatment of gastric ulcers (Adnan et al. 2014). The 10% ethanolic extract of *C. arabica* showed anti-gastric ulcer activity (Zakaria et al. 2002).

Ethanol (EtOH) is one of the most often utilized models for assessing the gastroprotective efficacy of natural products (Ali et al. 2023; Ali et al. 2024a, b). Ethanol use creates an imbalance between pro-oxidant and antioxidant mediators, hence producing oxidative stress. Gastric tissue MDA rises in this condition whereas decreased glutathione (GSH) (Taha et al. 2012; Barboza et al. 2018; Eraslan et al. 2020; Yoldaş et al. 2022). Oxidative stress activates the nuclear factor kappa B (NF-κB) transcription factor, which then generates more inflammatory mediators including interleukin-6 (IL-6) and tumor necrosis factor alpha (TNF-α) (Takahashi et al. 2001; Peng et al. 2014). Substance P (SP) is a member of the tachykinin (TK) peptide family. Earlier research showed that SP and its receptor G protein coupled receptor (NK-1R) might contribute to the development of gastric mucosal damage in rat and pig models **(**Xu et al. 2018; Zalecki 2019**)**. It has been underlined that activation of NF-κB and translocation into the nucleus to raise the production of inflammatory cytokines might follow activation of NK-1R by SP **(**Douglas and Leeman 2011).

Therefore, the current study was performed to evaluate the gastroprotective impact of AR-B in ethanol-induced gastric injury in rats via modulating oxidative stress/SP/NK-1R/NF-κB loop.

## Results

### Characterization of Arabincoside B

The structure of AR-B was elucidated using ^1^H and ^13^C NMR (Abdel-Sattar et al. 2022). Refer to supplemental materials: refer to Table S1 for ^1^H and ^13C^ NMR data (400, 100 MHz, respectively) and Figs. S1, S2, and S3 or the structure, ^1^H, and ^13^C NMR spectra of Arabincoside B.

### Justification of the selected doses

The selection of the Arabincoside B (AR-B) dose was based on results of a pilot study done involving ethanol-induced gastric injury and previous studies. There was no significant difference observed between the doses of 50 and 75 mg/kg of arabinoside B (El-Shiekh et al. 2023). In addition, the doses of 25 and 50 mg/kg of russelioside B, a very closely related pregnane glycoside, were selected and demonstrated a significant difference (Abdel-Sattar et al. 2018; El-Shiekh et al. 2021).

### The effect of Arabincoside B on pro-inflammatory cytokine level

**Fig. 1:**
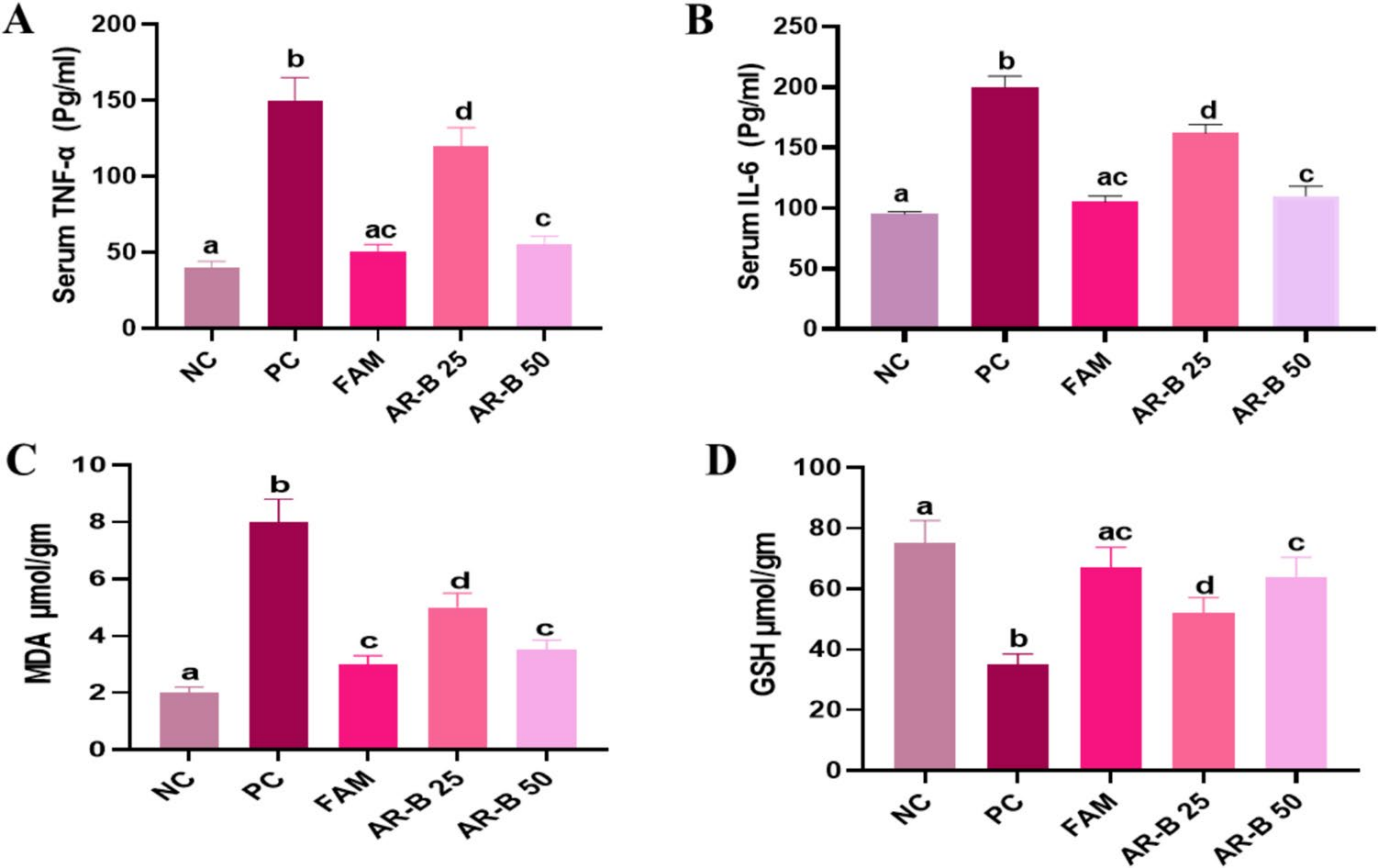
Impact of pretreatment with AR-B at doses of (25 or 50 mg/kg) on serum TNF α (**A**), IL-6 (**B**) levels, stomach tissue MDA (**C**) and GSH (**D**) concentrations in different experimental groups in ethanol-induced gastric injury rat model. Values are presented as mean (*n* = 6) ± SD, and statistical analysis was carried out using one-way ANOVA followed by Tukey’s post hoc multiple comparison test. Bars that are sharing a common superscript letter (a, b, c and d) are not significantly different, p<0.05. *NC* Normal Control, *PC* positive control, *FAM* famotidine, *AR-B 25* Arabincoside B (25 mg/kg), *AR-B 50* Arabincoside B (50 mg/kg)

Figure 1A and B depicts the variations in serum TNF-α and IL-6 levels across the different experimental groups. Untreated PC rats exhibited a significant increase in the serum TNF-α levels that were 3.7 times higher and serum IL-6 levels that were 2.3 times higher as compared to normal control rats. Proinflammatory factors (TNF-α and IL-6) were significantly reduced by 66.6 and 47.5% in the FAM-treated rats as compared to the PC rats, respectively. Nevertheless, no notable difference was detected between the FAM-treated group and the NC group. When compared to untreated PC rats, AR-B 25 significantly reduced serum levels of the latter two pro-inflammatory cytokines by 20 and 19%, respectively. Furthermore, compared to PC rats, AR-B 50-treated rats demonstrated a 63 and 45% reduction in the elevation of the aforementioned parameters, respectively. However, there was no discernible difference between the FAM- and AR-B 50-treated groups. These results revealed that there was a significant difference between the AR-B 25- and AR-B 50-treated groups with superior observed anti-inflammatory potential in the AR-B 50 pretreated rats.

### Effect of Arabincoside B on biomarkers of oxidative stress and antioxidant parameters in gastric mucosa

To assess oxidative stress, we measured MDA, as a marker of oxidative stress and a product of lipid peroxidation. As shown in Fig. 1C, the PC group showed a significant increase in MDA concentration in gastric mucosa, reaching fourfold higher than NC rats. Compared to the PC group, administration of FAM, AR-B 25, or AR-B 50 significantly decreased the gastric concentration of MDA by 62.5, 37.5 and 56.2%, respectively. It is worth mentioning that there is no unambiguous distinction between the rats treated with FAM and the AR-B 50 group.

The stomach mucosal GSH concentration in the PC group dropped significantly by 53.3% as compared to the NC group. Following the administration of FAM to the animals, the mucosal GSH concentration significantly increased by 47.7% as compared to the PC group, and it did not exhibit a significant difference from the NC group. The groups pretreated with AR-B 25 exhibited a 32.6% significant elevation in mucosal GSH concentration relative to the PC group. The AR-B 50 therapy led to a 45.3% significant augmentation in stomach GSH concentration in the rats, with no significant differences observed between the groups treated with FAM or AR-B 50 mg, Fig. 1D. These results pointed that there was a significant difference between the AR-B 25- and AR-B 50-treated groups with superior observed antioxidant potential in the AR-B 50 pretreated rats.

### Western blot analysis

#### Effect of Arabincoside B on P-NF-κB expression in gastric mucosa

**Fig. 2:**
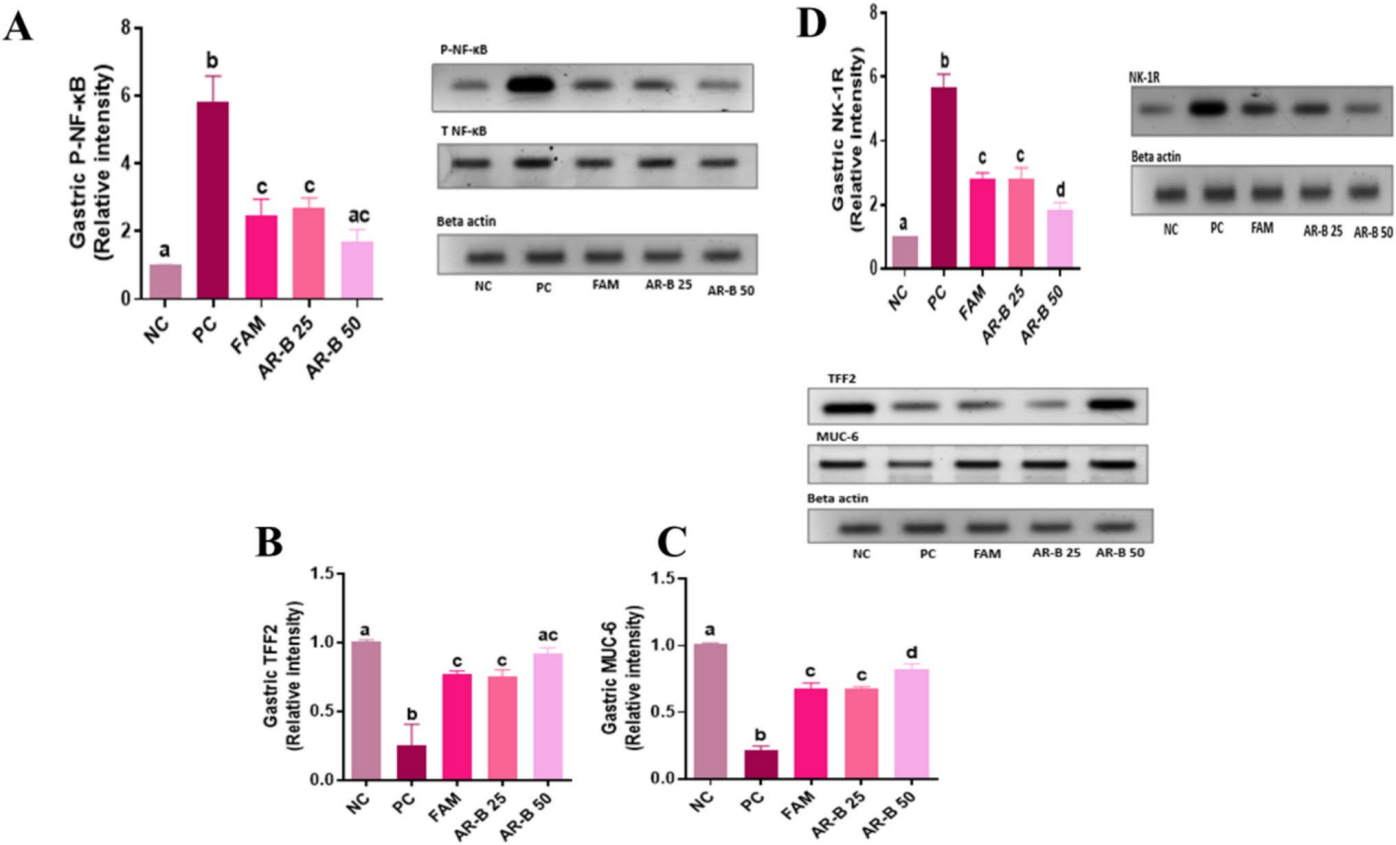
Impact of pretreatment with AR-B at doses of (25 or 50 mg/kg) on P-NF-κB P65 (**A**), TFF-2 (**B**), MUC-6 (**C**), and NK-1R (**D**) gastric mucosal expression in different experimental groups in ethanol-induced gastric injury rat model. Values are presented as mean (*n* = 3) ± SD, and statistical analysis was carried out using one-way ANOVA followed by Tukey’s post hoc multiple comparison test. Bars that are sharing a common superscript letter (a, b, c and d) are not significantly different, p<0.05. *NC* Normal Control, *PC* positive control, *FAM* famotidine, *AR-B 25* Arabincoside B (25 mg/kg), *AR-B 50* Arabincoside B (50 mg/kg)

As illustrated in Fig. 2A, P-NF-κB P65 expression levels were significantly increased in the PC group by 82.6% regarding the NC group. However, FAM, AB-R 25 mg, and AB-R 50 mg pretreatment significantly decreased P-NF-κB P65 protein expression by 57.5, 53.3 and 70.8%, respectively, compared to the PC group. There was no discernible significant difference between the NC and AR-B 50-treated groups. These findings demonstrated a significant difference between the AR-B 25- and AR-B 50-treated groups, with the AR-B 50 pretreatment rats exhibiting greater observed anti-inflammatory capability.

#### Effect of Arabincoside B on TFF-2 and MUC-6 protein expression in gastric mucosa

Gastritis PC rats showed a 75.3% significant decrease in TFF-2 protein expression as compared to the NC group of healthy rats, Fig. 2B. Treatment with FAM resulted in a 70.5% significant increase in the TFF-2 protein expression concentration, followed by AB-R 25 mg at 65.9% and AB-R 50 mg at 72.2%, in comparison to the gastritis rats in the PC group. It was essential to demonstrate that there are no significant differences among the rats treated with AR-B 50 mg and NC groups. In the same way, the MUC-6 protein expression in the gastric mucosa of PC with gastritis significantly diminished by 78.7% in comparison to healthy rats in the NC, Fig. 2C. Rats treated with FAM, AR-B 25 mg, and AR-B 50 mg exhibited significant increases in MUC-6 protein expression, reaching 68.1, 68.1, and 70.3%, respectively, in comparison to the PC group. These findings showed that the groups treated with AR-B 25 and AR-B 50 differed significantly, with the rats pretreated with AR-B 50 exhibiting better gastroprotective potential.

#### Effect of Arabincoside B on NK-1R expression in gastric mucosa

Figures 2D illustrates the alteration in NK-1R expression in gastric mucosa of different experimental groups. Untreated PC rats had a protein expression concentration 5.6-fold greater than that of NC rats. The expression concentration of NK-1R protein was significantly diminished by 49.9, 50.6, and 67% in the FAM-, AR-B 25-, and AR-B 50-treated rats, respectively, compared to the PC rats. Moreover, AR-B 50-treated rats exhibited the most significant drop in NK-1R protein expression concentration compared to another treated group including AR-B 25-treated rats.

#### Effect of Arabincoside Bon Ulcer index in macroscopic examination of gastric tissue

**Fig. 3:**
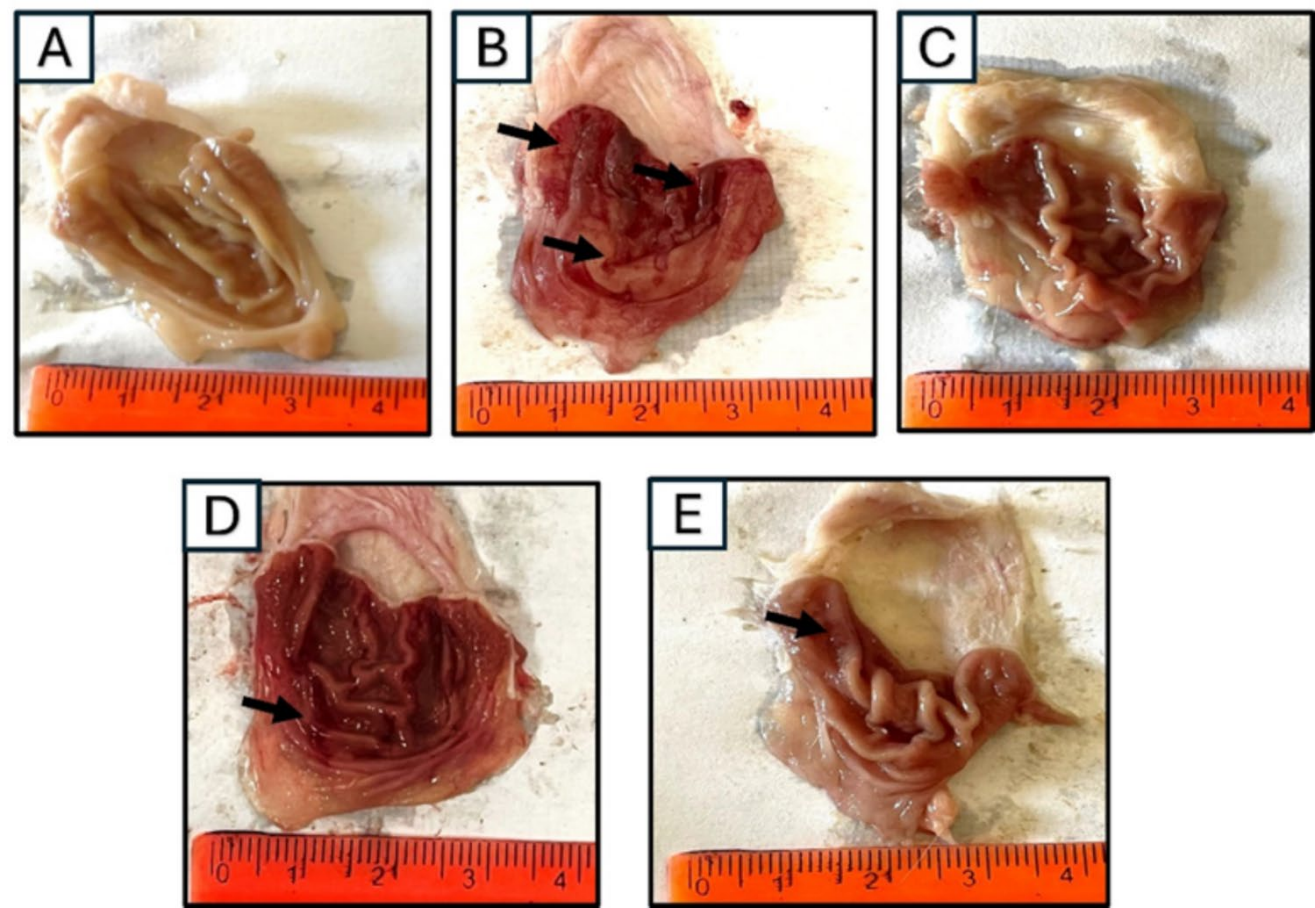
Macroscopic examination of gastric tissues of different experimental groups in ethanol-induced gastric injury model in rats (scale bar = 1 cm). Black arrows point at the linear ulcers. **A** Normal control (NC) showed pale pink glistening gastric mucosa. **B** Positive control (PC) showed dusky red congested mucosa with longitudinal ulcers (black arrows). **C** Famotidine-treated group (FAM.) showed a marked protective effect. **D** Arabincoside B (25 mg/kg) (AR-B 25) group showed a moderate protective effect with residual congestion and ulcers (black arrow). **E** Arabincoside B (50 mg/kg) (AR-B 50) group showed intact mucosa with focal ulcers (black arrow)

Gastric tissue of the normal group showed pale pink mucosa showing rugae covered by a glistening intact mucosal surface. The positive control showed dusky red congested mucosa with evident longitudinal ulcers and areas of punctate hemorrhage. They occupied 27% of gastric mucosa. Meanwhile, FAM showed a notable protective effect where the gastric mucosa was intact and glistening with no ulcers detected. The AR-B (25 mg/kg) group showed a moderate protective effect. Some longitudinal ulcers were seen, and they occupied 15% of the mucosa. The protective effect was augmented in the AR-B (50 mg/Kg) group where ulcers were only focally seen, and they occupied 7% of the mucosa as shown in Fig. 3.

### Histopathologic assessment of H&E-stained gastric tissues

**Fig. 4:**
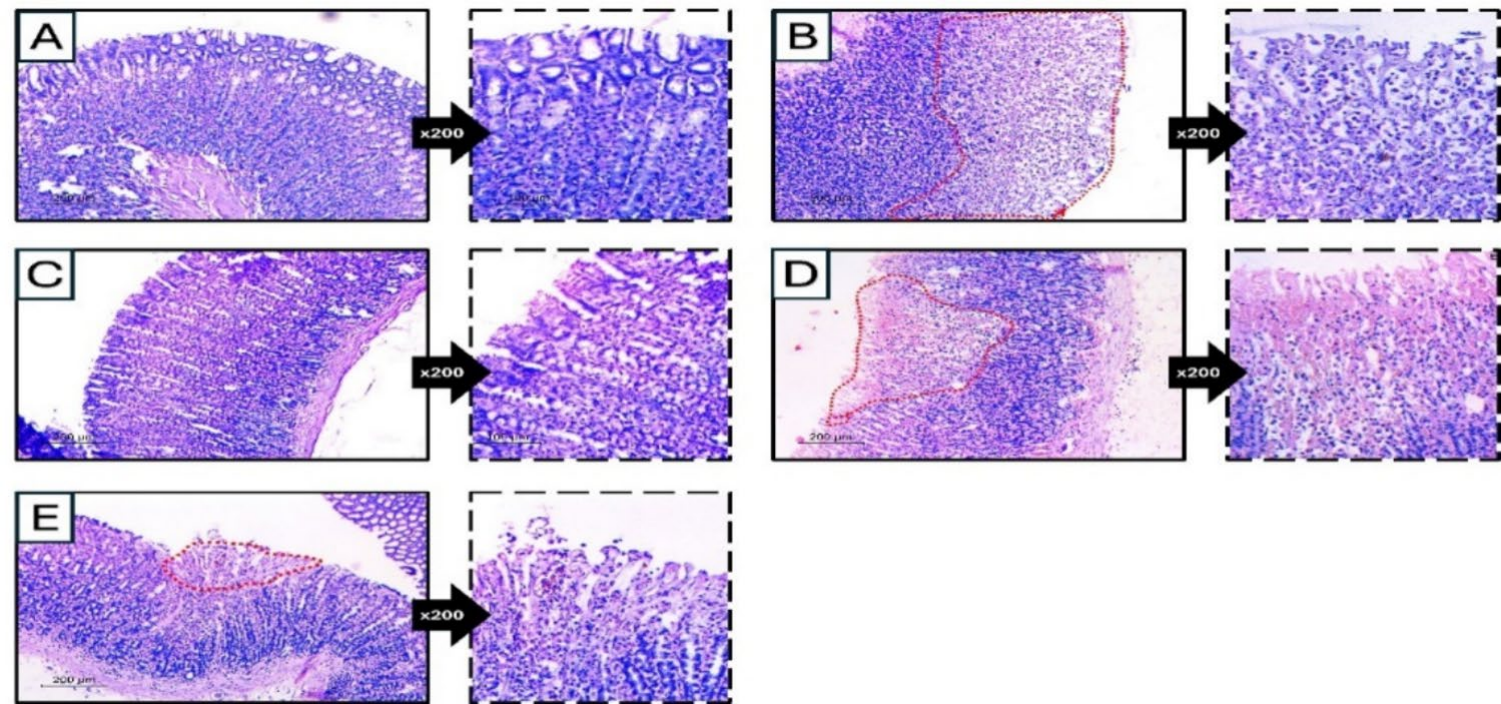
Histopathologic assessment of H&E-stained gastric tissues of different experimental groups in an ethanol-induced gastric injury model in rats. **A** Normal control (NC) showed intact gastric mucosa. The gland was long tubular arranged in an ordered manner, and high power showed gastric pits lined by viable columnar cells. **B** The positive control (PC) showed wide areas of coagulative necrosis (dashed area). High power showed necrotic sloughed cells and inflammatory cell infiltration. **C** Famotidine-treated group (FAM) showed marked protective effect. The gastric mucosa was intact, and high power showed viable mucosa. **D** Arabincoside B (25 mg/kg) (AR-B 25) pretreated group showed moderate protective effect with residual area of necrosis (dashed area). High power showed superficial zone of coagulative necrosis and mild inflammation. **E** Arabincoside B (50 mg/kg) (AR-B 50) pretreated rats showed a minimal superficial coagulative necrosis. High power showed focal coagulative necrosis and minimal residual inflammation (low power x100, high power x 200)

The normal group showed gastric wall covered by intact mucosa (Fig. 4). It was formed of long tubular glands arranged in ordinal manner. They were lined by columnar cells with basal ovoid nuclei and pale cytoplasm. No edema or inflammation was detected in the lamina propria. In contrast to positive control which showed wide areas of epithelial coagulative necrosis. The necrotic cells showed dense eosinophilic cytoplasm and dense pyknotic nuclei. They were sloughed in most areas. The lamina propria was edematous with moderate to severe inflammation. The FAM was able to protect the gastric mucosa against the injurious effect of ethanol. The gastric mucosa was intact with only mild edema. Meanwhile, AR-B had a moderate protective effect when it was supplied in a 25 mg/kg dose. The areas of coagulative necrosis were decreased with mild to moderate inflammation and edema, while it had a better protective effect on the AR-B 50 mg/kg dose. Only small superficial coagulative necrosis was seen with minimal inflammation and edema. A score for gastric injury was assessed out of 10. The score for group (PC) was 9 as compared to group (N), which was 0, while the score for the different experimental groups pretreated with FAM, AR-B 25, and AR-B 50 was 1, 6, and 4, respectively.

### Mucus secretion assessment by PAS staining

**Fig. 5:**
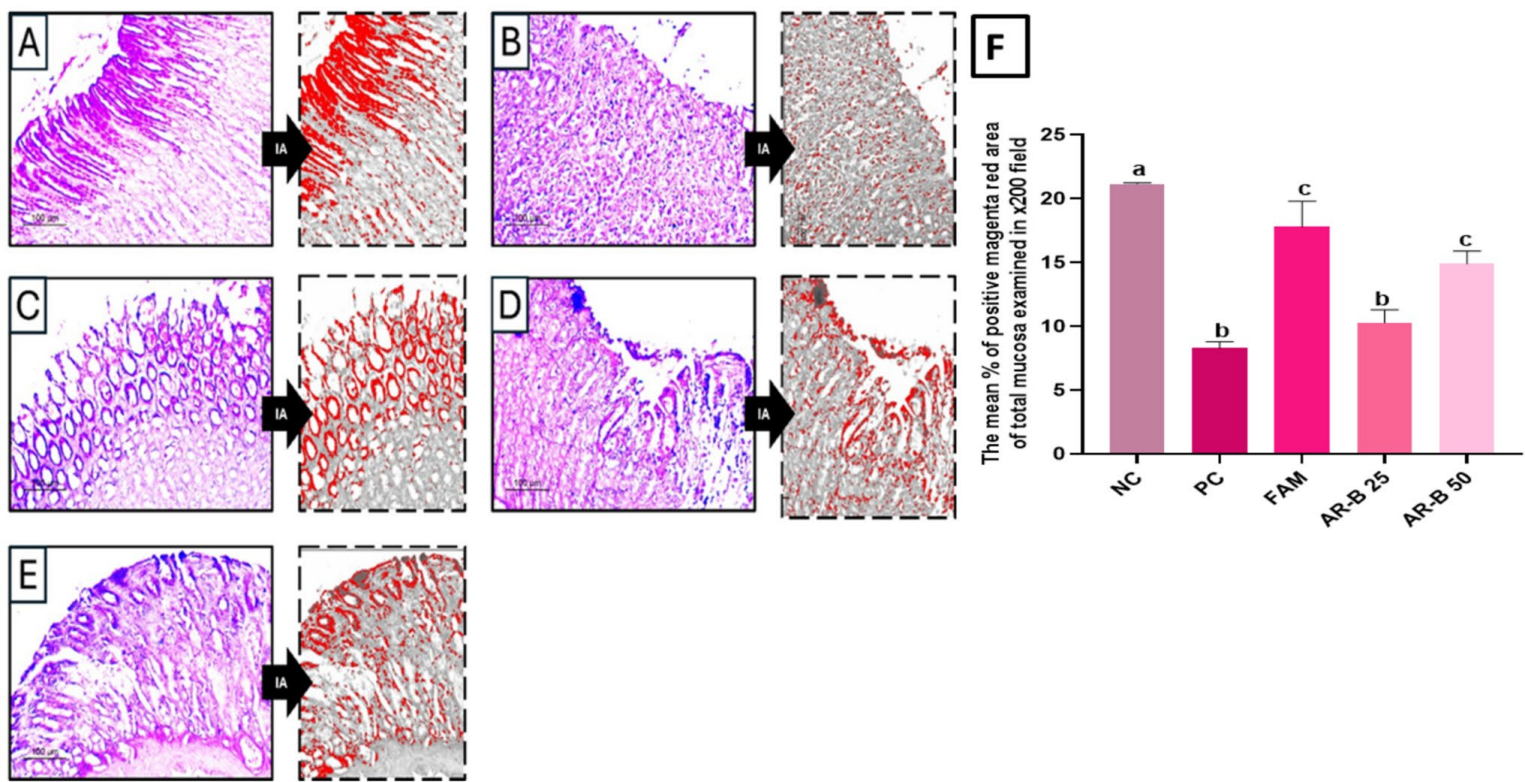
Mucus secretion assessment in Periodic acid Schiff (PAS)-stained sections of gastric tissue of different experimental groups in ethanol-induced gastric injury model in rats. **A** Normal control (NC) showed preserved mucus cell population. **B** Positive control (PC) showed marked mucus depletion. **C** Famotidine-treated group (FAM) showed near normal restoration of mucus secretion. **D** Arabincoside B (25 mg/kg) (AR-B 25) group showed mild protective effect. **E** Arabincoside B (50 mg/kg) (AR-B 50) group showed the best effect. PAS photo (×200) and its analyzed photo by image analysis software highlighting positive PAS areas in red color. **F** A bar chart highlighting the mean percentage of positive magenta red area of total mucosa examined in ×200 field in each experimental group

PAS staining was used to assess mucus secretion in normal group superficial parts of gastric glands; they were showing a positive magenta red signal highlighting mucus-secreting cells, Fig. 5. Near total depletion was seen in the positive control. Near total restoration of mucus secretion was seen in FAM-treated rats. The AR-B was able to partially restore mucus secretion in dose-dependent pattern in 25 and 50 mg/kg doses. The mean percentage of positive magenta red area of total mucosa examined in a x200 field was 21.16 for normal rats and 8.3 for gastric injured rats, while the aforementioned percentage was 17.8, 10.3, and 14.9 for FAM, AR-B 25, and AR-B 50 pretreated rats, respectively.

### Immunohistochemical assessment of substance P expression in gastric tissues

**Fig. 6:**
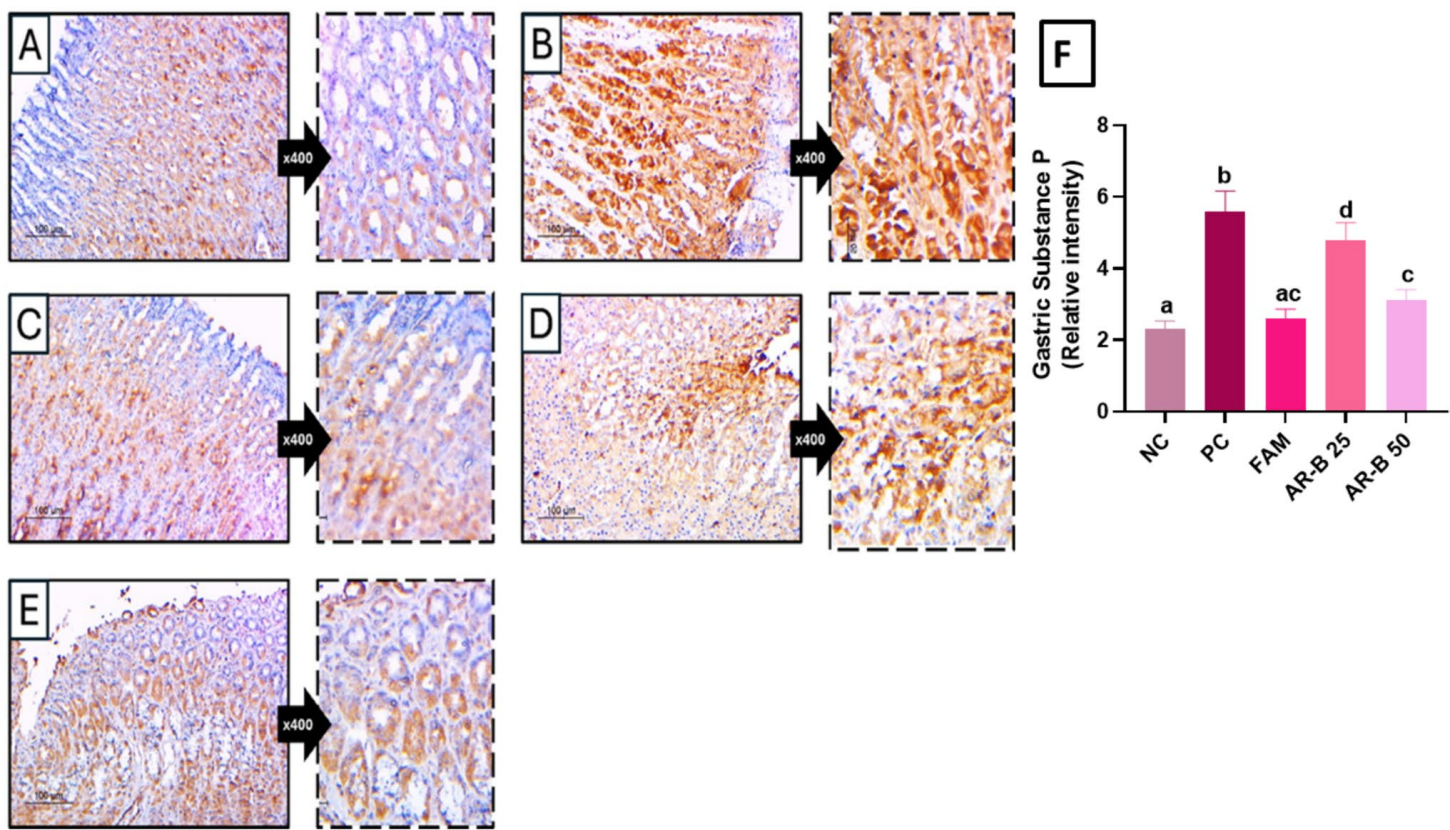
Immunohistochemical assessment of substance P expression in gastric tissues of different experimental groups in ethanol-induced gastric injury model in rats. **A** Normal control (NC) showed weak staining in mucosal crypts epithelium. **B** Positive control (PC) showed strong cytoplasmic staining. **C** Famotidine-treated group (FAM) showed weak staining. **D** Arabincoside B (25 mg/kg) (AR-B 25) group showed moderate protective effect. **E** Arabincoside B (50 mg/kg) (AR-B 50) group showed better effect. Substance P-IHC (×200) and high power (×400). **F** A bar chart highlighting the gastric substance P relative intensity in each experimental group

In normal group, few cells in deep crypts showed faint brown staining. In contrast to positive control which showed strong staining in wide areas of gastric glands in mucosa adjacent to ulcers indicating increased substance P secretion. The staining decreased markedly in famotidine-treated rats. Arabincoside B was able to partially decrease substance P expression in dose-dependent pattern in AR-B 25 and AE-B 50 mg/kg doses as shown in Fig. 6.

The substance P immunohistochemical expression optical density in the gastric mucosa of PC with gastritis was 2.3 as compared to 5.6 of healthy rats in the NC, while rats pretreated with FAM, AR-B 25 mg, and AR-B 50 mg demonstrated 2.6, 4.8, and 3.1, respectively.

### Statistical correlation

Table 1 illustrates the statistical correlations between MDA, SP, NK-1R, and P-NF-κB within the AR-B 50 pretreated group using the Spearman coefficient test. The results of the statistical correlations revealed a positive correlation between MDA, SP, NK-1R, and P-NF-κB within the AR-B 50 pretreated group.

**Table 1:** Statistical correlations between MDA, SP, NK-1R and NF-B within the AR-B 50-treated group.

	MDA	SP	NK-1R
P-NF-κB	rs **=** 0.778283, *p* < 0.001	rs **=** 0.773175, *p* < 0.01	rs **= **0.578162, *p* < 0.05

## Discussion

The stomach mucosal tissue injury is a hallmark of gastric ulcer, which is a serious common gastrointestinal disease. Even though there are numerous drugs available to treat stomach ulcers, they all have a number of negative effects, and stopping a prescription might lead to relapses (Ali et al. 2024a, b).

Multiple species of *Caralluma* have demonstrated cytoprotective or ulcer-healing activities. Notable examples include *C. arabica* (Zakaria et al. 2002), *C. adscendens* var. fimbriata (Tambe et al. 2010), *C. flava* (Raees 2018), *C. tuberculata* (Alharbi et al. 1994), *C. attenuata* (Garg et al. 2016), *C. quadrangula* (Ibrahim et al. 2023) and *C. penicillata* (Albaser et al. 2014). El-Shiekh et al. (2023) examined (AR-B)’s ability to resist oxidative stress and suppress inflammation in an LPS mouse model (El-Shiekh et al. 2023). In addition, the anti-inflammatory potential of AR-B has been shown by promoting wound healing in a rat model making it a potential veterinary wound dressing (Ali et al. 2024a, b).

The aim of the present study was to assess the gastroprotective effect of AR-B, a pregnane glycoside from *C. arabica*, in ethanol-induced gastric injury in rats via altering oxidative stress/SP/NK-1R/NF-κB loop.

There are many models for induction of gastric injury, pylorus ligation, or substances that injure or necrotize the mucosa, such as ethanol, acetic acid, or NSAIDs (e.g., indomethacin) (Simões et al. 2019).

Ethanol-induced gastric injury is one of the most often utilized models for assessing the gastroprotective efficacy of natural products (Ali et al. 2023; Ali et al. 2024a, b). One explanation for this is that excessive alcohol use was thought to be one of the main causes of GU in people as well as being a fast and reproducible model of peptic ulcer (Guzmán-Gómez et al. 2018). It has been demonstrated that ethanol could induce gastric injury via causing dehydration, disruption of the gastric mucosal membranes, cytotoxicity, the recruitment of leucocytes that exacerbate inflammation, oxidative stress, decreased gastric blood flow, and the release of mucus and bicarbonate (Ali et al. 2023; Ali et al. 2024a, b). Furthermore, compared to other GU models such as pylorus ligation and stress, the ethanol-induced GU model has an advantage as the aforementioned lesions have hemorrhage, desquamation of epithelial cells, infiltration of inflammatory cells, and substantial submucosal edema, which are significant traits displayed by the acute form of peptic ulcer in humans (Fu et al. 2021); however, the sole use of ethanol-induced gastric injury limits the study of chronic ulcer development and healing processes. Moreover, this model is not suitable for evaluating agents that target acid-related ulcerogenesis (Raish et al. 2021). Originally, A. Robert et al. employed the ethanol model in 1979 to specifically demonstrate the cytoprotective action of prostaglandins (Robert et al. 1979).

It has been illustrated that both TFF-2 and MUC-6 played a crucial role in forming a gel layer that protects the stomach’s mucous membranes (Yeo et al. 2021). Previous studies highlighted the ability of ethanol to decrease the expression of TFF-2 as well as halting the gene expression of MUC-6 in gastric ulcer in rats (Aziz et al. 2019; Shin et al. 2021). Our results confirmed the previous findings showing a significant decrease in the gastric tissue expression of both MUC-6 and TFF-2 in ethanol untreated rats. Moreover, AR-B at a dose of 50 mg/kg significantly promoted the expression of both MUC-6 and TFF-2, thereby preserving gastric mucous membranes more than AR-B at a dose of 25 mg/kg. These results were supported by a previous finding, which illustrated the efficacy of *C. arabia* in augmenting stomach prostaglandin and mucin production while reducing gastric acidity (Zakaria et al. 2002).

The use of ethanol may lead to oxidative stress, marked by an imbalance between free radical production and their neutralization, contributing to the pathophysiology of stomach damage (Ilhan et al. 2022). The final result of lipid peroxidation, known as MDA, indicated damage to the gastrointestinal mucosa and was consistently associated with GSH depletion. Our study demonstrated a significant effect of AR-B at a dose of 50 mg/kg in reducing MDA and elevating GSH levels in gastric mucosa tissues more than that observed with AR-B at a dose of 25 mg/kg. This finding aligns with earlier research that emphasized the role of AR-B in reducing oxidative stress in various rat models (El-Shiekh et al. 2023; Ali et al. 2024a, b).

Oxidative stress status can increase the production of inflammatory cytokines through the activation of the NF-κB transcription factor (Zhou et al. 2023; Elbaz et al. 2024) The mucosa lining the stomach might get damaged due to an excess of inflammatory cytokines and free radicals. The pro-inflammatory cytokines TNF-α and IL-6 played a vital role in promoting neutrophil infiltration into inflamed areas of the stomach and hindering the healing of gastric ulcers (Mousa et al. 2019). The rats in the PC group showed higher levels of phosphorylated NF-κB P65 in the stomach tissues and higher serum TNF-α and IL-6 levels compared to the control group. The results of our study were in agreement with other studies (Ercan et al. 2020; Ibrahim et al. 2024). A notable decrease in serum levels of TNF-α and IL-6, as well as the downregulation of P-NF-κB p65 protein expression in gastric tissues, was observed in AR-B pretreated rats, with superior effect of AR-B 50 more than AR-B 25. The AR-B anti-inflammatory action was previously highlighted in earlier studies (Chen et al. 2009; El-Shiekh et al. 2023).

In numerous cases of persistent inflammation of the intestines, levels of substance P and NK-1R were found to be significantly elevated (Mantyh et al. 1988, 1989). In addition, previous research had shown that SP is essential for inflammation pathogenesis because it activates NF-κB, which in turn increases the transcription of genes that promote inflammation (Bardelli et al. 2005; Koon et al. 2005). Our investigation found that rats pretreated with ethanol alone had higher stomach expression of SP and NK-1R. Pretreatment of rats with FAM, AR-B 25, or AR-B 50 considerably decreased SP and NK-1R expression; however, rats treated with AR-B at a dosage of 50 mg/kg showed the greatest reduction. This study is the first to show that AR-B inhibits the SP/Nk-1R expression in ethanol-induced gastric injury rat model.

Based on these findings, it appears that AR-B may have a gastroprotective function due to its anti-inflammatory and antioxidant properties. One potential rationale for the gastroprotective effect is that the antioxidant characteristics reduce the production of pro-inflammatory cytokines by stopping the activation of NF-κB. Decreases in SP and NK-1R expression also led to a gradual repression of NF-κB activation and inflammation. We also found a positive statistical link in the AR-B 50-treated group between MDA, SP, NK-1R, and NF-κB, which supports our findings.

Macroscopic analysis of excised stomach tissues from several experimental groups showed that the PC group had severe damage, with ulcers covering 27% of the mucosa as compared to the normal group with healthy gastric mucosa. This was consistent with the findings of a prior study (Chen et al. 2016). Famotidine was used as a standard drug. AR-B at a dose of (50 mg/kg) provided the best protection, with ulcers affecting just 7% of the mucosa. Histopathological examination of H and E-stained gastric tissue sections showed a healthy gastric mucosa with well-organized tubular glands and no signs of edema or inflammation in normal rats. In contrast, the positive control group showed significant epithelial necrosis, with necrotic cells and marked inflammation and edema in the lamina propria. All these histopathological changes were reverted by all given treatments superiorly with AR-B 50 pretreatment.

The PAS-stained gastric tissue sections showed that the normal group had strong mucus secretion, while the PC group had near-total depletion. Famotidine pretreatment nearly restored mucus secretion, and AR-B partially restored it in a dose-dependent manner. Previous research found that *C. arabica* extract increases gastric prostaglandin and mucin production and reduces stomach acidity (Zakaria et al. 2002; Albaser et al. 2014). Finally, our macroscopic, histopathological, and mucus content revealed by PAS stain results correlated with AR-B’s gastroprotective capabilities.

## Conclusion

The current investigation demonstrates the potential gastroprotective benefit of AR-B, particularly at a dosage of 50 mg/kg, against ethanol-induced stomach damage in rats. This was corroborated by a substantial increase in the gastric tissue expression of TFF-2 and MUC-6, which safeguard the gastric mucosa. Furthermore, the gastroprotective efficacy of AR-B may be attributed to its antioxidant and anti-inflammatory characteristics, as seen by the reduction of MDA, TNF-α, IL-6, and NF-κB levels, with an increase in GSH levels. The downregulation of (SP) and its receptor, (NK-1R), further diminishes inflammation. We may conclude that inhibiting oxidative stress and SP/NK-1R reduced NF-κB activation and decreased the generation of inflammatory cytokines.

## Supplementary Information

Below is the link to the electronic supplementary material.

Supplementary file1 (DOCX 433 kb)

## Data Availability

Not applicable.
