# Supplementary material for: Gastroprotective effect of Arabincoside B isolated from Caralluma arabica against ethanol-induced gastric injury via modulating oxidative stress/SP/NK-1R/NF-κB loop
Source: Inflammopharmacology. 2025 Aug 7;33(8):4859–69. doi: 10.1007/s10787-025-01885-w (PMC12396985; doi:10.1007/s10787-025-01885-w)
Supplement: Supplementary file 1 — Supplementary file1 (DOCX 433 kb) [file 10787_2025_1885_MOESM1_ESM.docx]

**Supplementary materials**

**Gastroprotective effect of Arabincoside B isolated from *Caralluma arabica* against ethanol-induced gastric injury via modulating oxidative stress/ SP/ NK-1R/NF-κB Loop**

**Dalia E. Ali^1^, Othman S. S. Al-Hawshabim^2^, Sarah A. Abd El-Aal^3^, Eman Sheta^4^**, **Sherihan Salaheldin Abdelhamid Ibrahim^5^, Amira A. El-Gazar^6^, Essam Abdel-Sattar^7^*, Ghada M. Ragab^8^**

^1^ Department of Pharmacognosy and Natural Products, Faculty of Pharmacy, Pharos University in Alexandria, Alexandria, Egypt

^2^ Department of Biology, Faculty of Science, University of Aden, Aden, Yemen

^3^ Department of Pharmacy, Al-Kut University College, Al Kut, Wasit 52001, Iraq

**^4^** Department of Pathology, Faculty of Medicine, Alexandria University, Alexandria, Egypt.

**^5^** Department of Pharmacology & Therapeutics, Faculty of Pharmacy, Pharos University in Alexandria, Alexandria, Egypt.

^6^ Department of Pharmacology and Toxicology, Faculty of Pharmacy, October 6 University, Giza 12585, Egypt.

^7^ Department of Pharmacognosy, Faculty of Pharmacy, Cairo University, Cairo, Egypt,11562.

**^8^** Department of Pharmacology and Toxicology, Faculty of Pharmacy, Misr University for Science and Technology, Giza 12585, Egypt.

***Corresponding author:**

https://orcid.org/0000-0002-0617-1420

**E-mail:** essam.abdelsattar@pharma.cu.edu.eg.

**Postal address:** Department of Pharmacognosy, Faculty of Pharmacy, Cairo University, El-Kasr El-Aini St, 11562 Cairo, Egypt.

[**Tel:** 23639307](Tel:23639307); **Fax:** +2023628426, **Cell phone:** +201065847211

**Co-Corresponding author:** Sherihan Salaheldin Abdelhamid Ibrahim

Department of Pharmacology and Therapeutics, Faculty of Pharmacy, Pharos University in Alexandria (PUA)

Postal address: Canal El- Mahmoudia Street, Smouha, Alexandria, Egypt

Phone number: + (203) 3877115

E-mail: ( [Sherihan.abdelhamid@pua.edu.eg](mailto:%20Sherihan.abdelhamid@pua.edu.eg)) or (dr_sherri_is@hotmail.com)

ORCID: 0000-0003-3467-5172

Phone number: 00201063584747

**Abstract**

Gastric ulcer is a common gastrointestinal condition. Arabincoside B (AR-B), a pregnane glycoside isolated from the aerial parts of *Caralluma arabica*, shows multiple pharmacological effects. This study aimed to investigate the gastroprotective therapeutic effects of AR-B in ethanol-induced gastric injury in rats. Rats were divided as follows: Group I (NC): Received 1 mL/day of normal saline; Group II (PC): Received distilled water then 95% ethanol (1 mL per rat) after one hour; Group III (FAM): Received oral famotidine (20 mg/kg); Group IV & V (AR-B groups): Received 25 and 50 mg/kg of AR-B, respectively. Treatments (FAM or AR-B) were administered 1 hr prior to ethanol. The rats were sacrificed after 1 hr after ethanol administration. Gross inspections as well as histopathological assessment of stomach tissues of untreated ethanol only treated rats revealed major alterations as compared to that of normal rats. Pretreatment with AR-B showed enhancement in the gross, and histological alterations. Moreover, AR-B at the dose of (50mg/kg) possessed superior anti-inflammatory and anti-oxidant effects. This was confirmed by significant lowering of the serum IL-6 and TNF-α levels, decreasing p-NF-κB gastric expression, decreasing MDA, and increasing GSH gastric levels. Furthermore, AR-B caused significant increase in TFF-2 and MUC6 stomach tissues expression, preserving the gastric mucosa. Also, gastric expression of substance-P and NK-1R were also decreased, which participated in reduction of inflammation. The current research highlights the gastroprotective impact of AR-B especially at a dose of (50 mg/kg), suggesting its future role in preventing recurrence in peptic ulcer patients.

**Keywords** *Caralluma arabica; Arabincoside B; Gastric Ulcer; MUC-6; SP/NK-1R; Anti-inflammatory*.

**Material and Methods**

**Plant material**

In July 2020, fresh aerial parts of *Caralluma arabica* N.E.Br. [Syn. *Ceropegia arabensis* Bruyns, *Desmidorchis arabica* (N.E.Br.) Meve & Liede; *Crenulluma arabica* (N.E.Br.) Plowes] were collected in the Aqan region of the Al-Musaimir District, Lahej Governorate, Southern Yemen (13° 22′ 871′′ N, 045° 83′ 344′′ E). Dr. Othman S. S. Al-Hawshabi, Associate Professor of Plant Taxonomy and Flora at the Department of Biology at the Faculty of Science, Aden University, Yemen, collected and authenticated the plant specimens. Subsequently, the plant was fragmented into little pieces and allowed to desiccate in the shade. The Department of Biology of the University of Aden, Faculty of Science, acquired voucher specimen #5659.

**Isolation of arabincoside B**

The aerial components of *Caralluma arabica* (300 g) were extracted using ethanol (95%), adhering to a previously documented methodology **(Abdel-Sattar et al. 2022).** A segment of the resultant water fraction (8 g) underwent chromatographic separation on a flash silica gel 60 column utilizing a solvent system of DCM-MeOH-H2O (10:2:0.1). The procedure facilitated the extraction of component AR-B (450 mg) from fraction Fr-13 (1.03 g) via precipitation, yielding a white amorphous powder. The isolation was performed with flash silica gel 60 (Merck, particle size 230–400 mesh) and RP-C18 silica gel (40–63 μm; Merck).

**Animals**

The Sprague-Dawley Albino rats, weighing between 150-175 g, were obtained from the animal house at Pharos University's Faculty of Pharmacy in Alexandria, Egypt. Every rat had its own polypropylene cage with dust-free bedding, the perfect environment in terms of temperature, humidity, and sunshine. In addition, we supplied the required quantity of food and water. For general conditions, all rats were observed at least once daily. The experimental procedures were approved by the research ethics approval committee unit of Pharos University's Faculty of Pharmacy (PUA-REC-012). All procedures adhered to the guidelines set by the National Institutes of Health (NIH) for the care and use of laboratory animals, as well as the Animal Research Reporting of In Vivo Experiments (ARRIVE) guidelines.

**Experimental protocol and the ethanol-induced gastric lesion method**

The animals were divided into five groups, each containing six rats (n = 6). Before conducting the ulcer experiment, the animals were fasted for 24 hours with unrestricted access to water. Two hours prior to the experiment and drug administration, water was removed from their cages. The selection of the arabincoside B (AR-B) dose was based on results of pilot study done involving ethanol-induced gastric injury and a previous study **(El-Shiekh et al. 2023)**. The rats were distributed randomly as follows: **Group I** (negative control) (NC): This group consisted of normal, healthy animals. **Group II** (positive control) (PC): The animals in this group received distilled water in the same volume as the AR-B given to the other groups. One hour later, they received 95% ethanol (1 mL per rat) **(Elshamy et al. 2020, Elshamy et al. 2020).** **Group III** (FAM-treated group): The animals in this group were given FAM (20 mg/kg orally) as a standard drug **(Bahadeen Aref et al. 2024).** **Groups IV** and **V:** Animals treated orally with AR-B (25, 50 mg/kg, respectively). Both the FAM and AR-B groups were treated one hour before orally consuming ethanol.

**Serum parameters**

At the end of the experiment, the animals were anesthetized with ketamine at a dosage of (100 mg/kg, IP) and xylazine at a dosage of (10 mg/kg, IP). Blood samples were obtained via cardiac puncture and then subjected to centrifugation at a speed of 3000 rpm for 10 minutes. This process resulted in the separation of clear serum, which was subsequently utilized for the measurement of TNF-α (Cat no. MBS825075, My BioSource, USA) and IL-6 (Cat no. ERA31RB, Thermo Scientific, USA) using ELISA kits, following the instructions provided by the manufacturer.

**Tissues preparation**

The excised gastric tissues were divided into three sets. The 1^st^ set was stored in 10% formalin for histological and immunohistochemical assessments, while the 2^nd^ set was utilized for the measurement of MDA (Cat no. MBS741034, My BioSource, USA), and GSH (Cat no. MAK364, Sigma Aldrich, USA) using ELISA kits, following the instructions provided by the manufacturer, and the 3^rd^ one for Western blot analysis.

**Western blot analysis**

Gastric tissue samples from each group were homogenized and lysed using RIPA buffer for protein extraction. Nuclear and cytoplasmic proteins were separated following the protocol provided in the Nuclear and Cytoplasmic Extraction Kit (Thermo Scientific, USA). The extracted proteins were resolved by electrophoresis on SDS-polyacrylamide gels (SDS-PAGE) and then transferred onto polyvinylidene fluoride (PVDF) membranes. The membranes were blocked for 2 hours with a 5% skim milk solution in TBST buffer. Primary antibodies were applied as follows: NF-κB (1:2000, ab16502, Abcam, UK), MUC-6 (1:1000, cat. no. 4814, Cell Signaling Technology, Inc.), TFF2 (1:1000, cat. no. 2859, Cell Signaling Technology, Inc.), NK-1R (1:1000, cat. no. 2859, Cell Signaling Technology, Inc.), and β-actin (1:1000, sc-47778, Santa Cruz, USA). This was followed by incubation with a secondary horseradish peroxidase-conjugated antibody. Immunolabeled proteins were visualized using ECL substrate, and their gray densities were quantified. Target protein levels were normalized to β-actin levels for standardization.

**Macroscopic examination:**

After sacrifice, gastric tissues were excised immediately and opened along greater curvature. Gastric juices were washed by saline and dried over a filter paper. The cleaned gastric mucosa was photographed using a digital camera (Leica Camera AG, Wetzlar, Germany) in good lightening condition. Gastric ulcer index and ulcer protective rate were calculated according to the equation **(Liu et al. 2024)**:

Ulcer index (%) = area of injured mucosa/total area of gastric mucosa × 100 %

**Histological assessments:**

Gastric tissues were fixed in 10% formalin for 24 hours. Serial sectioning of fixed tissues was done and strips were placed in plastic cassettes on their edges. Properly oriented tissues were dehydrated in ascending grades of alcohol, cleared in xylene and embedded in paraffin. Using a semi-automated microtome, five microns thick sections were cut and mounted on glass slides to be stained by hematoxylin and eosin (H&E) stain. Slides were examined by light microscopy by a pathologist blinded to the study groups. Evaluation of gastric injury was done by a scoring system out of 10. The score incorporated three parameters, edema (0-4) while each of coagulative necrosis and inflammatory cells infiltrate were graded (0-3) **(Balaky 2024)**.

**Mucus cell assessment**

Five microns thick sections were cut from previously prepared gastric tissue paraffin blocks and mounted on glass slides. Tissues were stained by periodic Acid Schiff (PAS) stain according to manufacturer protocol (Skytech laboratories, USA, # PAS-IFU). Mucus cells were stained magenta red. Using Image J software, the colours splitted and the positive magenta red area was assessed as % of total mucosa examined in x200 field. At least three fields were examined per rat and the mean was calculated.

**Immunohistochemical assessment**

Five microns thick sections were cut from previously prepared gastric tissue paraffin blocks. They were mounted on positively charged slides. They were stained by anti-substance P polyclonal antibody (#bs-0065R, rabbit polyclonal, BIOSS antibodies, USA) at 1:150 concentration. The staining was done using DAB technique by DAKO LINK48 autostainer. Slides were counterstained by hematoxylin and cover slipped to be examined under light microscope. Cells showing cytoplasmic brown staining were considered as positive. Assessment was done in viable areas within affected mucosa with avoidance of nonspecific staining in necrotic areas. Photos were taken at x200 and optical density of staining was measured using image J software **(Xu et al. 2018)**.

**Statistical analysis**

Statistical analysis was conducted using GraphPad Prism 8 (GraphPad Software Inc., San Diego, CA, USA). The gathered parametric data underwent analysis by a one-way ANOVA, succeeded by a Tukey post hoc test for multiple comparisons. We reported the results as the mean ± standard deviation from 6 distinct observations. The Spearman coefficient test was employed to assess statistical correlations among various factors using IBM SPSS software version 20.0 (Armonk, NY: IBM Corp). We considered p-values below 0.05 to be statistically significant.

**Table (1.S) ^1^H- and ^13^C-NMR spectral data of arabincoside B (DMSO-d_6_)**

|  | **^1^H-NMR** | **^13^C-NMR** |  | **^1^H-NMR** | **^13^C-NMR** |
| --- | --- | --- | --- | --- | --- |
| **No.** |  |  | **No.** | **Dig** | |
| 1 | 1.01 (1H, *m*), 1.80 (1H, *m*) | 37.18 | 1` | 4.20 (1H, *d*, *J*= 7.60) | 101.75 |
| 2 | 1.48 (2H, *m*) | 29.76 | 2` | 3.58 (1H, *m*) | 69.11 |
| 3 | 3.40 (1H, *m*) | 77.58 | 3` | 3.07 (1H, *m*) | 84.49 |
| 4 | 1.52 (1H, *m*), 2.35 (1H, *dd*, *J*= 2.84, 10.48) | 38.71 | 4` | 4.05 (1H, *b.s*) | 74.00 |
| 5 | - | 139.66 | 5` | 3.52 (1H, *m*) | 69.75 |
| 6 | 5.35 (1H,  *br d, J*= 5.24) | 122.19 | 6` | 1.13 (3H, *d*, *J*= 6.2) | 17.62 |
| 7 | 1.74 (1H, *m*), 2.16 (1H, *m*) | 27.40 | OCH_3_ | 3.38 (3H, *s*) | 58.22 |
| 8 | 1.58 (1H, *m*) | 37.14 | **Glc** | | |
| 9 | 1.10 (1H, *m*) | 45.89 | 1`` | 4.28 (1H, *d*, *J*= 7.72) | 103.50 |
| 10 | - | 36.98 | 2`` | 2.95 (1H, *m*) | 74.63 |
| 11 | 1.32 (1H, *m*), 1.43 (1H, *m*) | 20.82 | 3`` | 3.05 (1H, *m*) | 77.19 |
| 12 | 1.34 (1H, *m*), 2.12 (1H, *m*) | 38.85 | 4`` | 3.07 (1H, *m*) | 70.92 |
| 13 | - | 49.07 | 5`` | 3.31 (1H, *m*) | 76.82 |
| 14 | - | 84.62 | 6`` | 3.97 (1H, *d*, *J*= 11.64), 3.58 (1H, *m*) | 69.47 |
| 15 | 1.93 (1H, *m*), 1.61 (1H, *m*) | 33.85 | **Glc** | | |
| 16 | 1.83 (1H, *m*), 1.93 (1H, *m*) | 23.79 | 1``` | 4.36 (1H, *d*, *J*= 7.68) | 103.92 |
| 17 | 2.72 (1H, *dd*, *J*= 4.8, 9.2) | 62.99 | 2``` | 2.98 (1H, *m*) | 73.96 |
| 18 | 0.88 (3H, *s*) | 15.70 | 3``` | 3.05 (1H, *m*) | 77.19 |
| 19 | 0.93 (3H, *s*) | 19.66 | 4``` | 3.02 (1H, *m*) | 70.52 |
| 20 | - | 215.61 | 5``` | 3.10 (1H, *m*) | 77.36 |
| 21 | 2.20 (3H, *s*) | 31.94 | 6``` | 3.67 (2H, *dd, J*= 5.6, 11.60) | 61.54 |

**
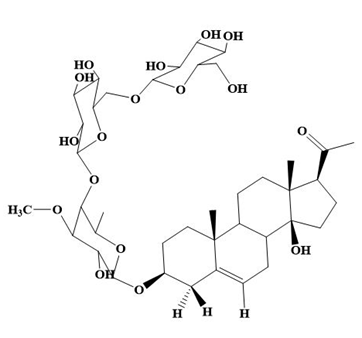
**

**Fig (1.S) Chemical structure of arabincoside B.**

Arabincoside B

**Fig (2.S) ^1^H-NMR spectrum of arabincoside B (DMSO-d_6_)**

Arabincoside B

**Fig (3.S) ^13^C-NMR spectrum of arabincoside B (DMSO-d_6_)**

**Reerences**

Abdel-Sattar, E. A., O. S. Al-Hawshabi, A. A. Shalabi, A. M. El Halawany and M. R. Meselhy (2022). "Arabincosides AD, pregnane glycosides isolated from Caralluma arabica." Tetrahedron 119: 132858. DOI: <https://doi.org/10.1016/j.tet.2022.132858>

Bahadeen Aref, B., S. Fakhri, M. R. Morovati, M. Ghanbari-Movahed, E. Mohammadi Noori, S. Miraghaei and M. H. Farzaei (2024). "Evaluating the Protective Effects of Aqueous and Hydroalcoholic Extracts of Plantago major Leaf in a Rat Model of Ethanol-induced Peptic Ulcer." Journal of Medicinal Plants and By-Products 13(3): 605-613. DOI: <https://doi.org/10.22034/JMPB.2023.362108.1548>

Balaky, S. T. J. (2024). "Anti H. pylori, anti-secretory and gastroprotective effects of Thymus vulgaris on ethanol-induced gastric ulcer in Sprague Dawley rats." Plos one 19(1): e0287569.

El-Shiekh, R. A., G. Nabil, A. A. Shokry, Y. H. Ahmed, O. S. Al-Hawshabi and E. Abdel-Sattar (2023). "Arabincoside B isolated from Caralluma arabica as a potential anti-pneumonitis in LPS mice model." Inflammopharmacology 31(3): 1437-1447. DOI: <https://doi.org/10.1007/s10787-023-01159-3>

Elshamy, A. I., N. M. Ammar, H. A. Hassan, S. L. Al-Rowaily, T. I. Ragab, A. E.-N. G. El Gendy and A. M. Abd-ElGawad (2020). "Essential oil and its nanoemulsion of Araucaria heterophylla resin: Chemical characterization, anti-inflammatory, and antipyretic activities." Industrial crops and products 148: 112272. DOI: <https://doi.org/10.1016/j.indcrop.2020.112272>

Elshamy, A. I., A.-R. H. Farrag, S. H. Mohamed, N. A. Ali, T. A. Mohamed, M. M. Menshawy, A. W. Zaglool, T. Efferth and M.-E. F. Hegazy (2020). "Gastroprotective effects of ursolic acid isolated from Ochrosia elliptica on ethanol-induced gastric ulcer in rats." Medicinal Chemistry Research 29: 113-125. DOI: <https://doi.org/10.1007/s00044-019-02465-8>

Liu, H., Y. Chen, Y. Hu, W. Zhang, H. Zhang, T. Su, J. Wang, Z. Yin, X. Zhao and X. Zhou (2024). "Protective effects of an alcoholic extract of Kaempferia galanga L. rhizome on ethanol-induced gastric ulcer in mice." Journal of Ethnopharmacology 325: 117845. DOI: <https://doi.org/10.1016/j.jep.2024.117845>

Xu, Y., J. Jia, C. Xie, Y. Wu and W. Tu (2018). "Transient receptor potential ankyrin 1 and substance P mediate the development of gastric mucosal lesions in a water immersion restraint stress rat model." Digestion 97(3): 228-239. DOI: <https://doi.org/10.1159/000484980>
